# Validation of an ELISA assay for measurement of the metabolite of serotonin, 5‐hydroxyindole acetic acid (5‐HIAA), in canine urine

**DOI:** 10.1111/jsap.70123

**Published:** 2026-03-25

**Authors:** D. Castillo, A. Swallow, T. Williams, P. Watson

**Affiliations:** ^1^ Department of Veterinary Medicine University of Cambridge Cambridge UK; ^2^ North Shore Veterinary Hospital Sydney New South Wales Australia

## Abstract

**Objectives:**

Serotonin (5‐hydroxytryptophan), implicated in a number of canine diseases, has a very short half‐life in the serum. Urine concentration of its breakdown product 5‐hydroxyindole acetic acid after an 8 hour fast is a more reliable measure of circulating serotonin in humans. This study aimed to validate a commercially available ELISA assay to measure 5‐hydroxyindole acetic acid concentrations in canine urine by comparing the analytical performance with the gold standard of liquid chromatography‐tandem mass spectrometry.

**Methods:**

Urine was collected from 26 dogs undergoing routine diagnostic investigations at one referral centre and rapidly processed and stored prior to testing for 5‐hydroxyindole acetic acid using the two methods. Deming regression and Bland–Altman analyses were used to compare the results between the two methods.

**Results:**

The ELISA demonstrated acceptable precision and repeatability (coefficient of variation <20%). There was good agreement between the two methods (bias 0.92 μmol/L; 95% limits of agreement −6.44 to 8.29 μmol/L), although the ELISA was not tested at values close to the upper‐end of the claimed analytical measurement range limit. The ELISA was likely to be very reliable at low concentrations but may exceed acceptable error limits at high concentrations.

**Clinical Significance:**

A commercially available ELISA was validated to measure urine 5‐hydroxyindole acetic acid. This is a less invasive method than blood sampling for serotonin and should give a more reliable indication of long‐term serum serotonin concentrations. More studies of normal and diseased dogs are needed to confirm these findings before applying them in a clinical setting.

## INTRODUCTION

Serotonin (5‐HT) is a biogenic amine, similar to adrenaline, noradrenaline and histamine, with multiple biologic roles (Mohammad‐Zadeh et al., 2008). Whilst free circulating 5‐HT is the active metabolite, levels are normally very low as most 5‐HT is contained within platelets (Yabut et al., 2019). Clearance involves monoamine oxidase (MAO), which breaks down 5‐HT to 5‐hydroxyindole aldehyde on the mitochondrial surface. This is metabolised to 5‐hydroxyindoleacetic acid (5‐HIAA) by aldehyde dehydrogenase, which is excreted in urine. Clearance is rapid; most exogenously administered 5‐HT being excreted as 5‐HIAA within 24 hours (Mohammad‐Zadeh et al., 2008; Yabut et al., 2019).

In dogs, 5‐HT has been implicated in the pathogenesis of myxomatous mitral valve disease (MMVD) in various breeds including Cavalier King Charles spaniels (CKCS) (Arndt et al., 2009; Cremer, Kristensen, & Reimann, 2015; Cremer, Moesgaard, et al., 2015; Ljungvall et al., 2013; Mangklabruks & Surachetpong, 2014; Oyama et al., 2020; Oyama & Levy, 2010) and also in pulmonary hypertension with mitral valve disease (Tangmahakul et al., 2021) and dilated cardiomyopathy (Fonfara et al., 2014). Several studies focussing on MVD have demonstrated increased circulating 5‐HT in CKCS compared to other breeds, although there is significant variation between studies and a large overlap in the reported 5‐HT concentrations (Arndt et al., 2009; Cremer, Kristensen, & Reimann, 2015; Cremer, Moesgaard, et al., 2015; Ljungvall et al., 2013).

Because of the short plasma half‐life, spot blood samples do not represent longer term concentrations. Both feeding and stress affect plasma 5‐HT concentrations in dogs (Tangmahakul et al., 2021; Wright et al., 2012). In humans, measurement of the urinary metabolite 5‐HIAA after an overnight fast in the home environment is the gold standard for diagnosis of serotonin‐secreting tumours, providing a more reliable indicator of 5‐HT concentrations over a longer period of time (Subash et al., 2022). Two previous studies have measured urine 5‐HIAA in dogs using gas chromatography mass spectrometry (Some & Helander, 2002) and high performance liquid chromatography with electrochemical detection (Christiansen et al., 2019). However, these techniques are expensive and not widely available. To facilitate future research into the role of serotonin in canine diseases, a simple, non‐invasive, reliable and accessible test of 5‐HIAA in the urine is needed. To achieve this, we aimed to validate an ELISA assay to measure canine urinary 5‐HIAA concentrations and compare the analytical performance with the current reference technique, liquid chromatography‐tandem mass spectrometry assay (LC–MS) (Grant et al., 2021; Miller et al., 2010).

## MATERIALS AND METHODS

Canine urine samples were obtained at the Queen's Veterinary School Hospital (QVSH), University of Cambridge, UK between November 2019 and November 2021. Urine creatinine concentration was measured in some dogs. Written owner consent was granted at the time of admission to the hospital. The study received ethical approval from the Department of Veterinary Medicine, University of Cambridge, UK  approval number CR247. Dogs undergoing diagnostic investigations that required a urine sample collection for urinalysis, irrespective of breed, age or health status, were included in the study. Only one sample per dog was included. Samples were taken at any time of day, at an unknown time after eating and from hospitalised patients. Samples were protected from light by using covered, 5 mL urine collection pots and were delivered to the laboratory immediately after collection *via* cystocentesis for immediate processing as a standard urinalysis. Up to 5 mL of urine was centrifuged at a relative centrifugal force (RCF = × *g*) of 358 for 5 minutes at room temperature. A total of 1.0 or 1.5 mL of excess supernatant was separated into two or three aliquots of 500 μL, respectively, and stored at −80°C until batch analysis of 5‐HIAA.

A commercially available ELISA testing kit (Ref: BA‐E‐1900, Immusmol) which uses a microtiter plate format was selected for the quantitative measurement of 5‐HIAA in urine (using the manufacturer's protocol). In brief, the methylated analyte in the standards, controls, patient samples and the solid phase‐bound analyte compete for a fixed number of antibody binding sites. After reaching equilibrium, the free antigen and free antigen–antibody complexes are removed by washing. The antibody that is bound to the solid phase is detected using an anti‐rabbit IgG peroxidase conjugate with 3,3′,5,5′‐tetramethylbenzidine (TMB) as a substrate. The absorbance is then read using a microplate reader at 450 nm. A standard curve was generated for each analytical run by plotting the absorbance readings against the corresponding standard concentrations using four parameter logistic regression. 5‐HIAA concentrations from patient samples were then interpolated from the standard curve.

LC–MS was utilised as the gold standard comparative method in this study. Urine 5‐HIAA analysis was performed by LC–MS (Laboratory Research Services, Addenbrooke's Hospital, Cambridge, UK) following solid phase extraction (SPE) of 5‐HIAA from urine. Urine is mixed with the internal standard containing deuterated 5‐HIAA and the mixture is applied to a SPE plate. After washing, 5‐HIAA is eluted with methanol. Following a dilution, the extracted samples are loaded onto a pentafluorophenyl (PFP) liquid chromatography column and the eluate is infused into the tandem mass spectrometer. Multiple reaction monitoring (MRM) is used to quantitate the 5‐HIAA against a standard curve by comparing the peak area ratio between 5‐HIAA and the deuterated internal standard. (Grant et al., 2021).

Prior to validation, the goal for acceptable performance was defined as a total allowable error of ±8 μmol/L at a concentration of ≤40 μmol/L and ±20% at a concentration >40 μmol/L based on the Royal College of Pathologists of Australasia allowable limits of performance in biochemistry (The Royal College of Pathologists of Australasia Quality Assurance Programs, 2021).

The replication experiment of the ELISA was performed to evaluate the imprecision or variation expected in a test result under the normal operating conditions of the assay. Two levels of known concentrations (measured by LC–MS) of urinary 5‐HIAA were measured to obtain estimates of random analytical error or imprecision, expressed as the coefficient of variation (CV) within‐run and between‐run. Five replicate measurements were performed at each known concentration level on the same analytical run to estimate the within‐run imprecision. To estimate the between‐run imprecision, replicate measurements were obtained from three different analytical runs on separate days. To avoid repeated freeze–thaw effects on urine samples used for evaluating the between‐run imprecision, urine samples of known concentrations were separated into three aliquots of 150 μL. The total imprecision was also calculated from all results for each concentration.

The method comparison experiment was performed to estimate the systematic difference or accuracy of the ELISA based on the differences observed between the two methods. The correlation coefficient (*r*) was calculated to determine whether ordinary linear regression will provide reliable estimates of errors between the methods, using a cut‐off of *r* = 0.975 (Westgard et al., 2020). Based on the coefficient of correlation *r* = 0.96, Deming regression was used. By use of regression statistics, an estimate of systematic error was obtained, including constant and proportional error. A Bland–Altman plot was also used to compare the two measurement techniques. The total analytical error was calculated to judge acceptability of the ELISA relative to the predetermined total allowable error at concentrations of potential clinical relevance or interest. All statistical analyses were performed using commercially available software (GraphPad Prism).

## RESULTS

**FIG 1:**
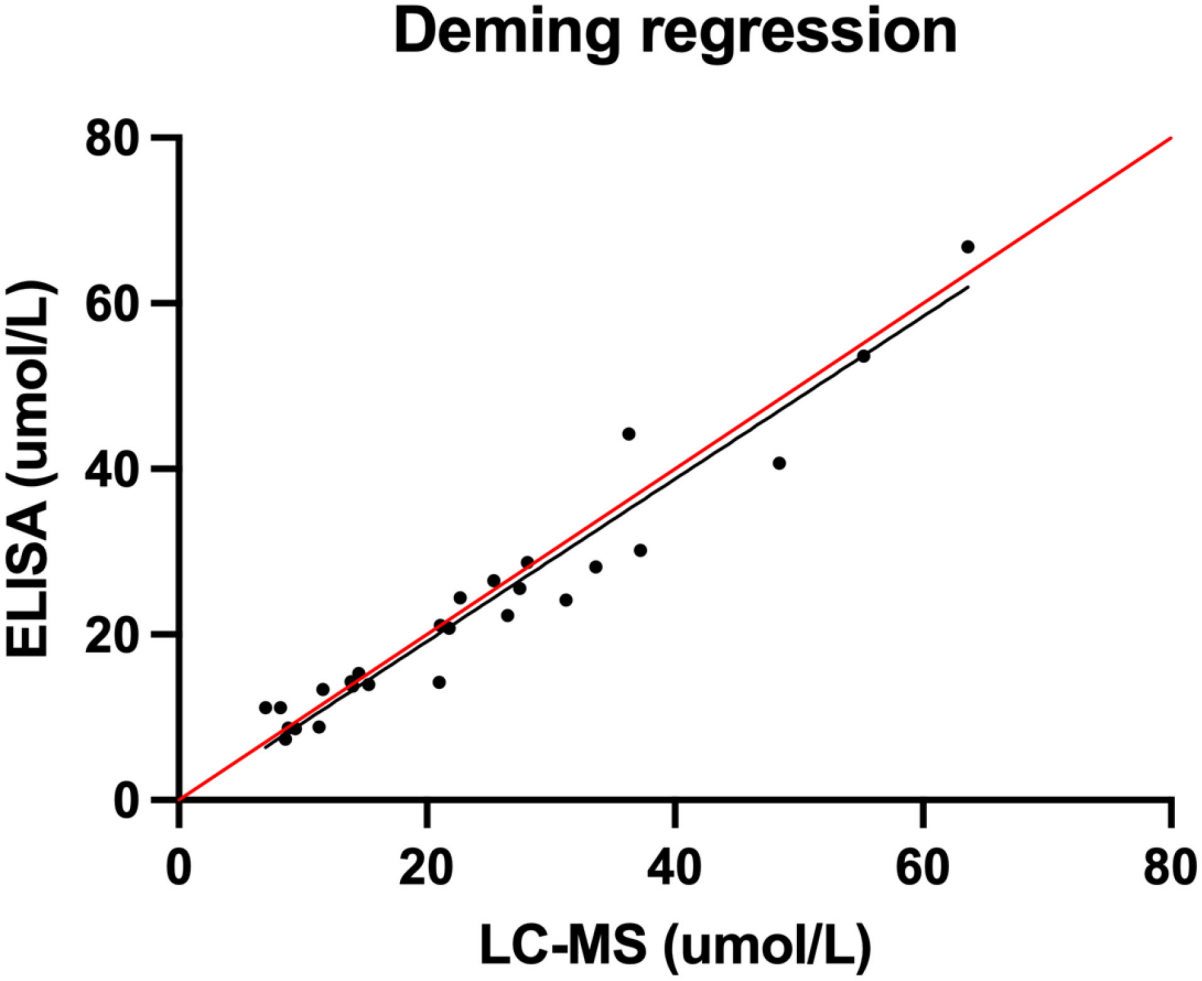
Deming regression plot. The regression line indicates good agreement between the methods. There is minimal constant and proportional error. The line of identity is shown in red.

**FIG 2:**
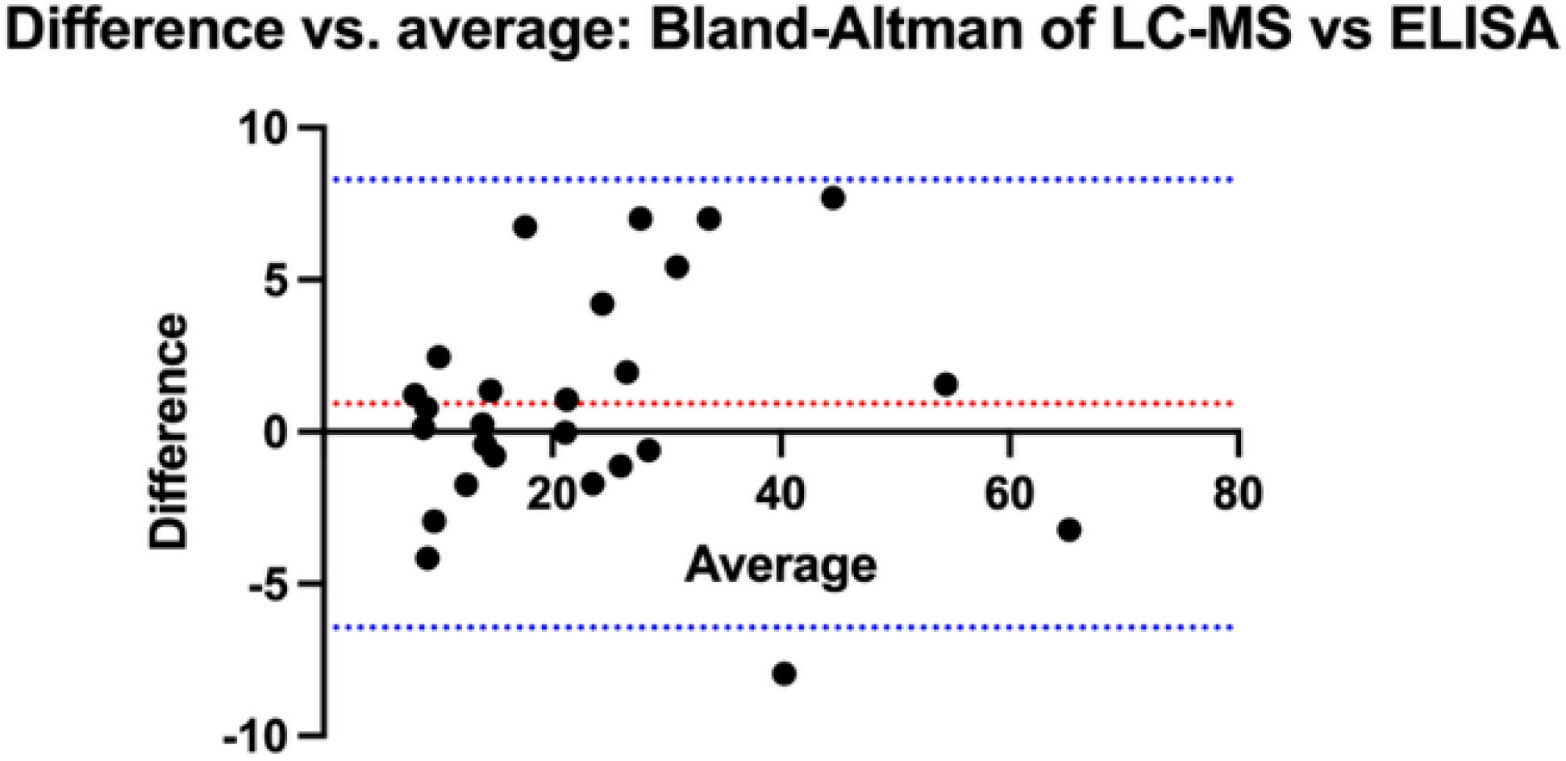
Bland–Altman plot. The difference between the line of equality (black line) and the mean of the differences (red dotted line) corresponds to the bias (0.92) between the methods. The distribution of the results was mostly within the limits of agreement which was defined as the mean difference ± 1.96 SD (blue dotted line).

The study population consisted of 26 dogs (6 males, 20 females) of different breeds which were referred to the referral hospital with various clinical presentations (Table 1). Urinary 5‐HIAA concentrations ranged from 7 to 63.6 μmol/L and 7.4 to 66.8 μmol/L, measured by LC–MS and ELISA, respectively (Table 1). Within‐run imprecision of the ELISA assay was 15.3% at low concentrations (9.6 μmol/L, Table 2) and 13.0% at high concentrations (55.2 μmol/L, Table 2). Between‐run imprecision was 13.0% and 10.1% at low and high concentrations, respectively (Table 2). The total imprecision was 19.3% and 11.3% at low and high concentrations, respectively (Table 2). Deming regression analysis demonstrated the presence of no significant proportional and constant error. The slope was 0.98 (95% CI, 0.82 to 1.13) and the y‐intercept was −0.49 (95% CI, −3.54 to 2.56) (Fig. 1). Bland–Altman analysis also demonstrated good agreement between the two methods (Fig. 2).

**Table 1:** Patient signalment, clinical history and urinary 5‐hydroxyindoleacetic acid (5‐HIAA) concentrations measured by liquid chromatography‐tandem mass spectrometry assay (LC–MS) and enzyme‐linked immunosorbent assay (ELISA). Urine 5‐HIAA to creatinine ratio is also shown in the 8 dogs in which urine creatinine measurements were available

Breed	Age	Sex	Clinical history	Urinary 5‐HIAA (umol/L) by LC–MS	Urinary 5‐HIAA (umol/L) by ELISA	Urine creatinine (mmol/L)	5‐HIAA by ELISA:UCR ratio
Cavalier King Charles Spaniel	6	Female, neutered	2‐3 m history of gagging whilst sleeping, marked lethargy. PUPD, polyphagia, weight gain (BCS 8/9)	48.4	40.7	12.048	3.37
Miniature schnauzer	1	Female, neutered	Previous urinary tract infection and struvite urolithiasis, currently on amoxiclav	22.7	24.4	N/A	N/A
Miniature schnauzer	4	Female, neutered	Pollakiuria	21	14.3	10.045	1.42
Labrador	8	Male, neutered	Polyuria, polydipsia, lethargy, inappetance	9.4	8.6	N/A	N/A
Collie cross	8	Female, neutered	Pollakiuria and stranguria	25.4	26.5	19.845	1.33
Alaskan Malamute	6	Male, neutered	Masticatory myositis on prednisolone	33.6	28.2	17.664	1.59
Pug	2	Female, entire	Porto‐systemic shunt	7	11.2	1.898	5.90
English Bull Terrier	6	Female, entire	Pollakiuria and haematuria	63.6	66.8	38.819	1.72
Cocker Spaniel	9	Female, entire	Haematuria	26.5	22.3	5.601	3.98
Miniature schnauzer	14	Male, neutered	Protein‐losing nephropathy	15.3	13.9	8.105	1.71
Fox Terrier	12	Female, neutered	Vomiting, jejunal lymphadenopathy	8.8	8.7		
Bichon Frise	10	Female, entire	Bladder stones	31.2	24.2		
Dachshund	11	Male, neutered	Diabetes mellitus and bladder stones	8.2	11.1		
Cross breed	12	Female, neutered	Chronic vomiting	55.2	53.6		
Cross breed	8	Male, neutered	Abdominal mass	14.5	15.3		
Cross breed	12	Female, neutered	Inappetance and weight loss	27.5	25.5		
Labrador	8	Male, neutered	Stranguria	37.2	30.2		
Staffordshire Bull Terrier cross	9	Female, neutered	Abdominal pain, hyporexia, diarrhoea	8.6	7.4		
Airedale Terrier	11	Female, neutered	Ascites	21.8	20.7		
Pug	2	Female, entire	Lumbar pain	36.3	44.3		
Labrador cross	4	Female, neutered	Porto‐systemic shunt	14	13.8		
Cocker Spaniel	3	Female, neutered	Previous urinary tract infection, intermittent diarrhoea	28.1	28.7		
Cavalier King Charles Spaniel	12	Female, neutered	Pancreatitis	11.6	13.3		
Cross breed	9	Female, neutered	Inappetance, weight loss, abdominal pain	13.9	14.3		
Pug	10	Female, neutered	Vomiting	11.3	8.8		
Cocker Spaniel	4	Female, neutered	Urinary incontinence and previous urinary tract infection	21.1	21.1		

**Table 2:** Within‐run and between‐run imprecision of urinary 5‐HIAA ELISA assay at low and high concentrations

	Replicate measurements (μmol/L)					Mean	SD	CV (%)
	1	2	3	4	5			
Within‐run imprecision								
Low concentration	18.40	18.90	13.14	15.70	19.23	17.07	2.60	15.3
High concentration	64.45	57.91	44.40	56.48	56.84	56.01	7.26	13.0
Between‐run imprecision								
Low concentration	14.29	13.77	11.14			13.07	1.69	12.9
High concentration	55.18	53.64	64.45			57.76	5.85	10.1

Total imprecision (combined within‐run and between‐run) was 19.3% and 11.3% at low and high concentrations, respectively.

CV, coefficient of variation; SD, standard deviation.

Based on data related to bias and assay precision obtained during assay validation, the total observed error at high and low concentrations of 5‐HIAA was calculated (bias + 2 * standard deviation (SD)). At high concentrations (55.2 μmol/L) the total observed error was 14.0 μmol/L (25.4%) which is greater than the 20% suggested maximum value for total allowable error at concentrations >40 μmol/L (Royal College of Pathologists of Australasia allowable limits of performance in biochemistry). At low concentrations (9.6 μmol/L) the total observed error was 3.7 μmol/L which is within the total allowable error goal of ±8 μmol/L at a concentration <40 μmol/L.

Urine creatinine concentrations were available in 8 dogs and their urine 5‐HIAA (ELISA) to creatinine ratios are shown in Table 1.

## DISCUSSION

This study validated a commercially available human ELISA for measurement of 5‐HIAA in urine in dogs by comparison with a gold standard method (liquid chromatography‐tandem mass). The test was found to be reliable except for a slightly higher observed error compared to acceptable error limits at higher concentrations.

The reported analytical measurement range of the ELISA is 0.9 μmol/L to 262.5 μmol/L. Although the lower and upper limits of the claimed measurement range were not verified, the goal was to obtain a wide range of results expected to be seen in the canine population. A previously published study of urinary 5‐HIAA excretion in fresh spot samples in a variety of species reported dogs to have similar concentrations to humans (5 to 40 μmol/L); although the canine values were not reported. In that study, the highest concentrations were found in hamsters (mean 89.3 μmol/L) and the lowest in rainbow trout (mean 2.0 μmol/L) and horses (2.3 μmol/L) (Some & Helander, 2002). In the current study, urinary 5‐HIAA in random samples from 26 dogs ranged from 7.4 to 66.8 μmol/L, consistent with this previous report; although it should be noted that the dogs in our study were presented to a referral centre, so they were not clinically normal. The more recent study of urinary 5‐HIAA in CKCS reported a 5‐HIAA to creatinine ratio (μmol/mmol) with values ranging from 1.3 to 3.7 (Christiansen et al., 2019). In the current study, urinary creatinine measurements were available in 8 dogs. The 5‐HIAA (ELISA) to creatinine ratios in these dogs were similar to this study, ranging from 1.33 to 5.9.

Samples close to the lower end of the claimed analytical measurement range limit were tested and performance was acceptable. Samples of high concentrations were also tested; however, they were not close to the upper end of the claimed analytical measurement range limit. Imprecision was also evaluated and was considered acceptable (CV < 20%), and specificity is presumed to be good, given that method comparison with LC–MS did not identify any significant constant error. Additional validation experiments such as spike recovery and dilutional parallelism were not performed in this study. Whilst these tests can provide further information regarding assay accuracy and the presence of interfering or cross‐reacting substances, the strong agreement observed between the ELISA and the reference LC–MS method across native canine urine samples suggests that clinically significant matrix interference was unlikely within the concentration range studied. These experiments should be considered in future validation work.

The ELISA method is a relatively low cost test and is a more accessible method for laboratories to use than LC–MS, both of which would make the test more widely available for veterinary clinicians than LC–MS. Further validation work should be considered in order to have an improved understanding of the different preanalytical factors that may affect measurement of 5‐HIAA concentrations. The important question remains as to whether measuring urinary 5‐HIAA will be a good surrogate marker for physiologically active circulating 5‐HT in dogs and therefore whether it will useful in studying disease pathogenesis. In the recently published study of CKCS, urine 5‐HIAA concentrations collected first thing in the morning after fasting showed no correlation with either serum or platelet poor plasma 5‐HT concentration (Christiansen et al., 2019). This calls in to question the reliability of either the blood 5‐HT assays or the urine 5‐HIAA assay. It is very possible that serum and plasma 5‐HT assays in previously published canine studies were inaccurate, which might explain some of the conflicting results. Differences between studies may be caused by differences in the methods used to assess circulating 5‐HT concentrations, the instability and transient circulation of free 5‐HT in the plasma or variations in important patient factors prior to analysis (pre analytical factors) including circadian rhythms and post prandial increases (Fukui et al., 2012). In addition, sample type, collection method, sample handling, sample stability or factors inherent to the study population such as age, breed, sex, health status, medications, stress or diet and timing in relation to feeding can all contribute to measurement variability and error (Alberghina et al., 2019; Tangmahakul et al., 2021; Wright et al., 2012). Different methodologies have been used to assess 5‐HT and its by‐products in canine platelet poor plasma, serum, platelets and urine (Arndt et al., 2009; Christiansen et al., 2019; Cremer, Moesgaard, et al., 2015; Ljungvall et al., 2013; Mangklabruks & Surachetpong, 2014). Many of the assays used for this purpose have not been validated in dogs, hence little is known about their performance characteristics, limitations and whether their methodology is fit for its intended use.

Most circulating serotonin is sequestered in platelets but free plasma serotonin is the active metabolite. Measurement of either plasma (free) serotonin or serum (free + platelet serotonin) are subject to a multitude of pre and post analytic factors already discussed. In human medicine, urinary and plasma 5‐HIAA are recognised as much more accurate markers of serum 5‐HT concentration than direct measurement of 5‐HT itself. (Yabut et al., 2019). Initially, 24 hour urine collection was recommended to allow for transient spikes in 5‐HIAA concentration, but urine collection in the morning after at least an 8 hour fast is strongly correlated with 24 hour collection and is now the standard test (Tellez et al., 2013). This method could be applicable in most dogs, with urine collected first thing in the morning at home and transported rapidly to the clinic in a similar way to other endocrine tests currently undertaken clinically such as urinary cortisol: creatinine ratios.

Future studies may benefit from verifying the analytical performance close to the upper end of the measurement range in addition to seeking to establish reference intervals and relevant medical decision limits to aid with interpretation of test results. Adjusting the total 5‐HIAA concentration for urinary specific gravity by relating it to the concentration of urine creatinine would be necessary in future clinical studies (Calanchini et al., 2019). In addition, it would be important to confirm that values after an 8 hour fast are as reliable as those obtained after 24 hour urine collection in dogs, as they are in humans.

In conclusion, we have validated an ELISA for measurement of 5‐HIAA concentration in the urine of dogs. This will provide a non‐invasive test of 5‐HT breakdown products which may help in the investigation of the role of 5‐HT in a multitude of canine diseases including gastrointestinal and fibrotic syndromes. Future studies are recommended in large groups of dogs of many breeds and disease states using urine samples collected first thing in the morning after an 8 hour fast to confirm the accuracy of this test and its usefulness in comparison to measurement of 5‐HT in serum or plasma. After further validation, measurement of 5‐HIAA in the urine after an 8 hour fast should provide a more accurate measurement of circulating serotonin concentration than spot blood samples, facilitating further research into its role in mitral valve disease in a number of breeds. It might be expected to replace serum tests of 5‐HT in the same way as measurement of urinary metanephrines has replaced blood sampling for adrenaline in the diagnosis of phaeochromocytoma in dogs. It could also help confirm or refute the suspicion that CKCS have higher serotonin concentrations than other breeds and elucidate its potential role in other diseases such as progressive fibrosis of the pancreas and kidney in the CKCS (Kent et al., 2016).

## Author contributions

**D. Castillo:** Data curation; formal analysis; methodology; investigation; writing – original draft; writing – review and editing. **A. Swallow:** Data curation; formal analysis; methodology; investigation; writing – original draft; writing – review and editing. **T. Williams:** Conceptualization; data curation; methodology; supervision; project administration; writing – review and editing; writing – original draft; investigation; formal analysis. **P. Watson:** Conceptualization; data curation; formal analysis; supervision; methodology; investigation; writing – original draft; writing – review and editing; funding acquisition; project administration.

## Conflict of interest

No conflicts of interest have been declared by any of the authors.

## Funding

This work was supported by a grant from the British Small Animal Veterinary Association (BSAVA) PetSavers (CRP 15 17).

## Data Availability

All data is in the tables in the paper.
